# Supplementary material for: protoSpaceJAM: an open-source, customizable and web-accessible design platform for CRISPR/Cas insertional knock-in
Source: Nucleic Acids Res. 2024 Jun 26;52(15):e68. doi: 10.1093/nar/gkae553 (PMC11347160; doi:10.1093/nar/gkae553)
Supplement: gkae553_Supplemental_Files [file gkae553_supplemental_files.zip › Supplementary_figures_20240429_with_legend.pdf]

Supplementary Figures and Tables for: **protoSpaceJAM: an open-source, customizable and web-accessible design platform for CRISPR/Cas insertional knock-in**

Duo Peng[1,*], Madhuri Vangipuram[1], Joan Wong[1], and Manuel D. Leonetti[1,*]

[1]Chan Zuckerberg Biohub, San Francisco, CA, 94158, USA.

SUPPLEMENTARY FIGURES

Supplementary Figure 1. Recoding and strand selection impacts the rate of payload integration. (A) Schematic illustration of HDR resolving without payload integration due to homology between the target genome and the donor in the cut-to-insert region (left). “Repair track” mutations to reduce homology by recoding the cut-to-insert region promotes payload integration (right). **(B)** Schematic illustration of strand choice impacting payload integration as a function of cut site position relative to the insertion site with ssODN donors. ssODNs template DNA repair via a synthesis-dependent strand annealing mechanism. In the illustrated example, the insertion site is located on the 5’ side of the cut site. In this orientation, the plus strand (right) is more favorable for payload integration compared to the minus strand (left). The favored orientation will be reversed when the payload is on the 3’ side of the cut site.

Supplementary Figure 2. gRNA weights and recoding exclusion zones near splice junctions. (A-B) Sequence logo plots showing conservation of sequences near splice junctions in human, mouse, and zebrafish, using 1373460, 719549, and 848492 exon-intron and intron-exon junctions extracted from all annotated transcripts. **(A)** Different position weights are assigned to gRNAs according to the level of sequence conservation in different regions of the splice junctions. **(B)** Around exon-intron junctions, recoding is strictly prohibited within 3 bp upstream and 6 bp downstream of splice sites. Similarly, in the case of intron-exon junctions, recoding is prohibited within 3 bp upstream and 2 bp downstream of splice sites.

Supplementary Figure 3. Fluorescent tagging in human induced pluripotent stem cells (hiPSCs) using protoSpaceJAM designs. Integration efficiencies of a split-GFP fluorescent payload across 34 human protein-coding genes for which top-scoring gRNAs were located at various cut-to-insert distances. The percentage of GFP-positive cells is measured by flow cytometry and serves as a read-out of knock-in efficiency.

SUPPLEMENTARY TABLES

Supplementary Table 1 (Related to Figures 2B, 3C, 4D and Suppl. Figure 3). gRNA and payload sequences for fluorescent tagging experiments. Percentage of fluorescence-positive cells measured by flow cytometry are reported.

Supplementary Table 2 (related to Figure 2C). gRNA, payload and genotyping primer sequences for all targeted genes. The rate of HDR for each gene is reported (characterized by deep sequencing of the targeted alleles).

Supplementary Table 3 (related to Figure 5). Sequences of all primer pairs tested.

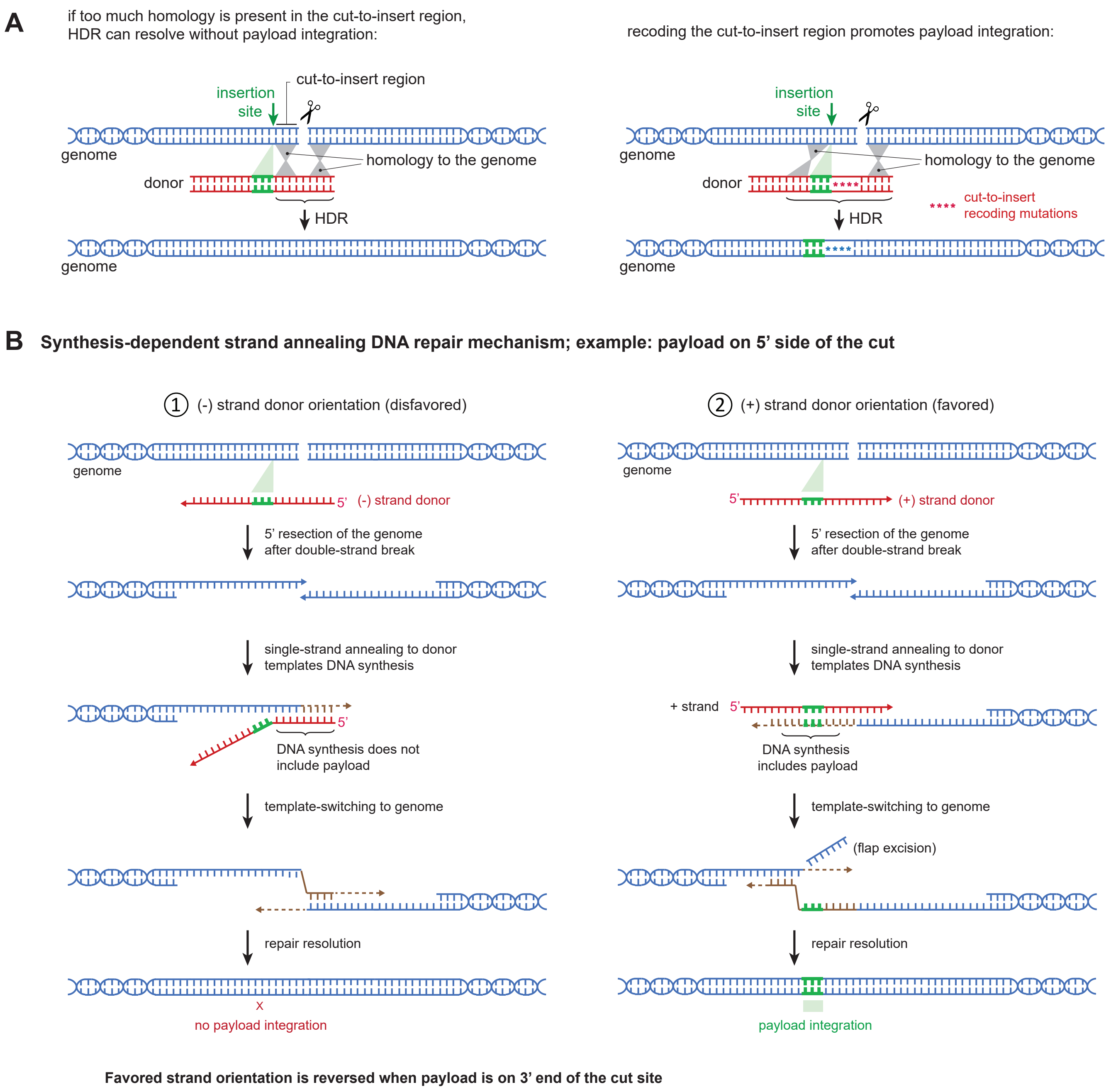

Suppl. Figure 1

Recoding and strand selection impacts the rate of payload integration

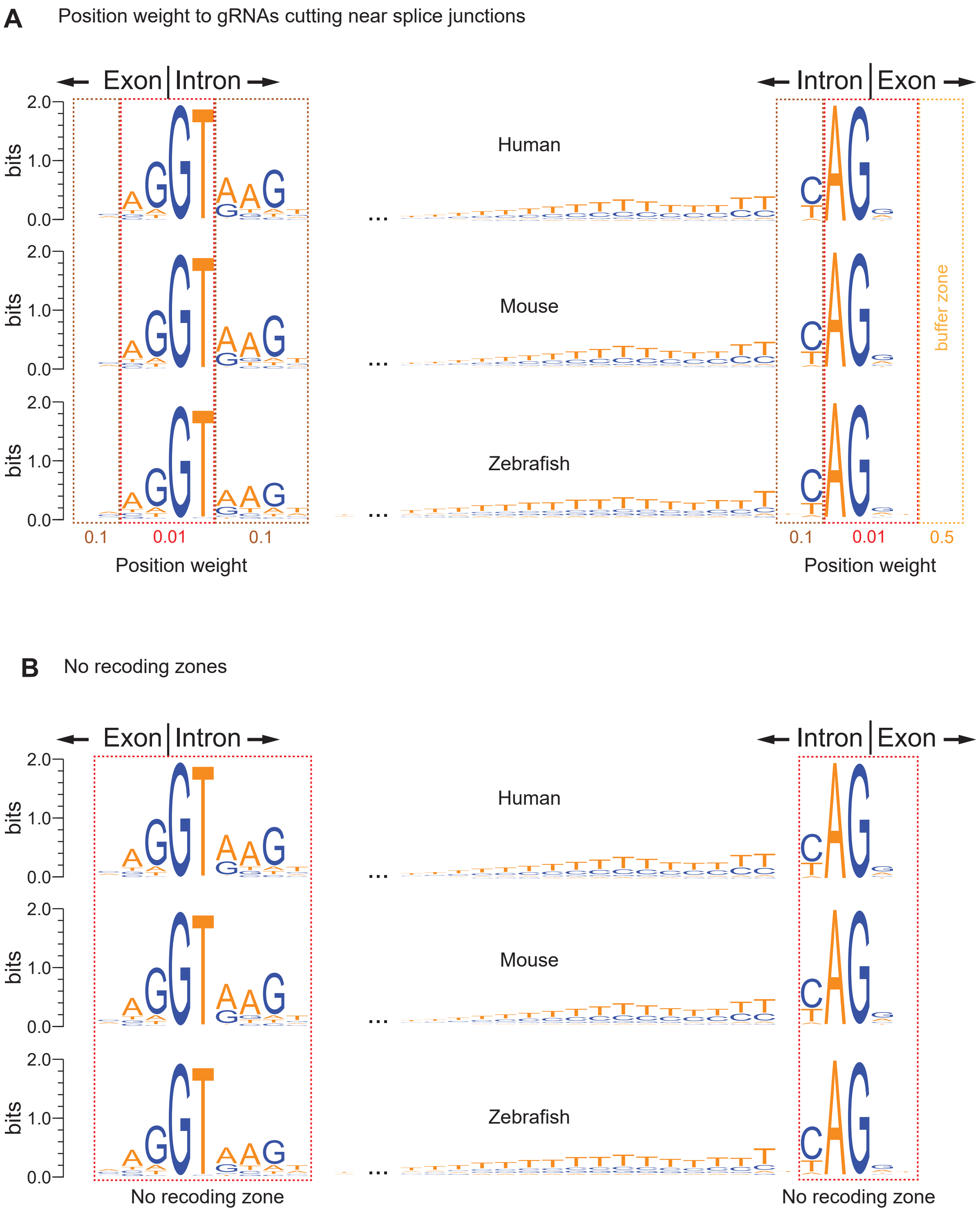

Suppl. Figure 2

Positional weighting to gRNAs that cleave near splice junctions while also avoiding recoding in proximity to these junctions.

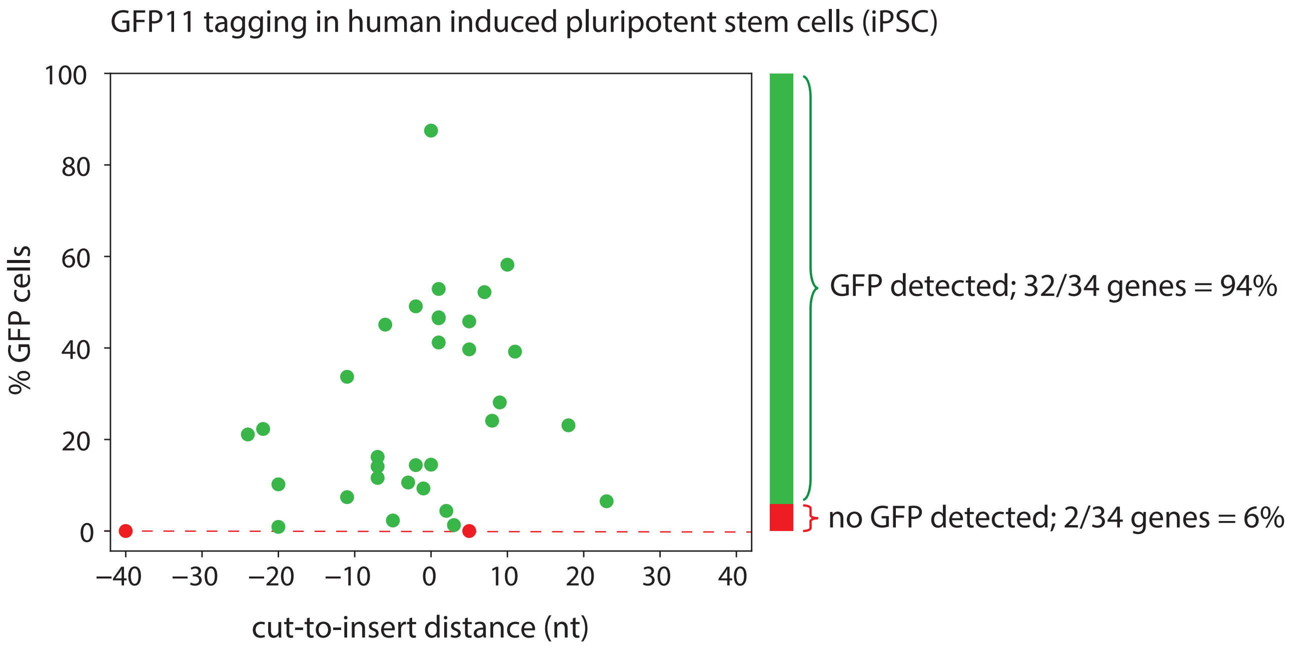

Suppl. Figure 3

Fluorescent tagging in human induced pluripotent stem cells (hiPSCs) using protoSpaceJAM designs.
